# Critical analysis of vestibular rehabilitation outcome according to dizziness etiology

**DOI:** 10.1016/S1808-8694(15)31172-1

**Published:** 2015-10-19

**Authors:** Roseli Saraiva Moreira Bittar, Maria Elisabete Bovino Pedalini, Jeanne Oiticica Ramalho, Ricardo Yoshimura

**Affiliations:** 1 PhD in Medicine, Assistant Physician at the Neurotology Ward - HCFMUSP; 2 PhD in Sciences, Speech and Hearing Therapist in charge of the Vestibular Rehabilitation Ward - HCFMUSP; 3 PhD in Sciences, collaborating physician - Neurotology Ward - HCFMUSP; 4 MD. Otorhinolaryngologist. Student of the Specialization course in Neurotology - FMUSP; Disciplina de Clinica Otorrinolaringológica do Hospital das Clínicas da FMUSP

**Keywords:** vestibular rehabilitation, dizziness

## Abstract

**Summary:**

Vestibular rehabilitation (VR) is an excellent therapy for dizziness patients. However, despite well managed, sometimes results are not suitable.

**Aim:**

evaluate VR outcome between patients according to dizziness etiology. Study design: Retrospective review of medical records. **Method**: Patients' records were analyzed between January 2002 and December 2004. As for inclusion criteria, patients should have had finished VR therapy and an established diagnosis. Patients were included in three VR outcome groups and compared according to etiology.

**Results:**

according to VR outcome 13 patients had no improvement, 24 had partial improvement and 22 complete improvement. The main etiologies were cervical syndrome, trauma, metabolic disorders, central nervous system disorders, anxiety and mood disorders, autoimmune disease and orthostatic intolerance. Patients with metabolic disorders showed better VR outcome than the others.

**Conclusion:**

VR therapy combined with clinical etiology treatment is an excellent approach for dizziness patients.

## INTRODUCTION

Body balance is a fundamental condition in the lives of people and when in disorder it brings high levels of stress to the patient and also difficulties to walk and guide oneself^1^. Any therapeutic proposal that aims at reestablishing body balance must take into account its influence in the daily life of the human being. As complementary therapy or only therapy, vestibular rehabilitation (VR) is an excellent therapeutic choice for patients with vestibular disorders. Besides reducing vestibular symptoms, VR also has a prophylactic intent, it helps reestablish the patients' trust in themselves, reduces stress and improves social relations and life quality^2^, ^3^, ^4^.

During the last six decades, after the first descriptions of the basic exercises used by Cawthorne & Cooksey, in soldiers that had suffered head injuries (HI), VR has been undergoing constant developments. Today, new approaches are being investigated, such as sensorial replacement and virtual reality in the therapy to recover body balance^5^,^6^. Primarily described for use in adults with post head injury deficits, today VR is used in children^7^ and in diseases where it was previously contraindicated, such as Meniere's disease (MD)^8^.

Notwithstanding, even when well applied, sometimes VR does not bring about the desired effects initially proposed. Some patients improve very little or almost nothing in terms of their symptoms, even if both the patient and the therapist are engaged.

## METHODS

This is a retrospective descriptive study of patients with body balance disorder referred for treatment in the Vestibular Rehabilitation Outpatient Ward, from January of 2002 to December of 2004. The study followed all the rules in effect for our institution, established by the Ethics Committee for the Analysis of Research Projects, authorized under protocol # 1027/03.

In order to select the sample we analyzed the charts of all the patients seen during this period in our ward. We excluded those with incomplete for follow up data, those who abandoned treatment and those who were suspended from treatment. Study variables were: etiological diagnosis of body balance disorder and clinical response to VR. The selected charts encompassed the patients who concluded their VR treatment.

In order to be admitted to VR, the patient had to be diagnosed in the classical neurotological assessment, including clinical history, otorhinolaryngological examination, cranial nerve assessment, laboratory work up, balance and coordination tests, electronystagmography, according to the routine established at the neurotology ward^9^.

The VR program was based on the global protocol first described by Cawthorne & Cooksey, later modified by Pedalini & Bittar^10^. The treatment was tailored according to the individual's needs, including adaptation or replacement exercises whenever necessary. The therapy sessions were developed in the outpatient ward and the patients were instructed to perform the exercises at home, twice a day and return monthly for reassessment. The final clinical evaluation of the treatment was made by means of a visual-analogue scale:

• remission: symptoms improvement greater than 70%.
• partial improvement: between 50 and 70% of symptoms improvement.
• no improvement: less than 50% of symptoms improvement.

The patients were classified in one of three groups according to their response to VR and they were compared among themselves as to the etiologies detected.

As far as etiology is concerned, it was considered of metabolic origin when there were metabolism disorders related to glucose alterations (diabetes, hyperinsulinemia, hypoglycemia), thyroidal (hyperthyroidism, hypothyroidism - including the so-called subclinical cases) or lipid alterations (increase in LDL cholesterol and triglycerides)9. It was considered to be of central origin when the patient had central vestibular disorders, cerebellar diseases, stroke, vertebro-basilar insufficiency and uncharacterized dysfunctions^11^. The so-called disorders of propioception, associated or not to structural lesions were classified as being of cervical etiology^12^. Etiology of trauma was considered in the dysfunctions stemming from local trauma or head injury, including those that started after otologic surgery (stapes, mastoidectomy, exeresis of glomus or vestibular schwannoma). Mood and stress disorders (MSD) encompassed all psychiatric disorders, including depression and panic. Auto-immune diseases (AID) were considered in cases of established diagnoses and in rheumatology follow up (vasculitis or thrombophilia). Orthostatic intolerance (OI) was considered in cases of diziness and vision darkening that happened when the person stood up quickly.

Statistical analysis included a case description design and the chi-squared test (c2) in order to compare the ratios of the many etiological proportions among the groups without improvement, with partial improvement or remission with VR, considering a significance value of p = 0.05.

## RESULTS

**Graph 1:**
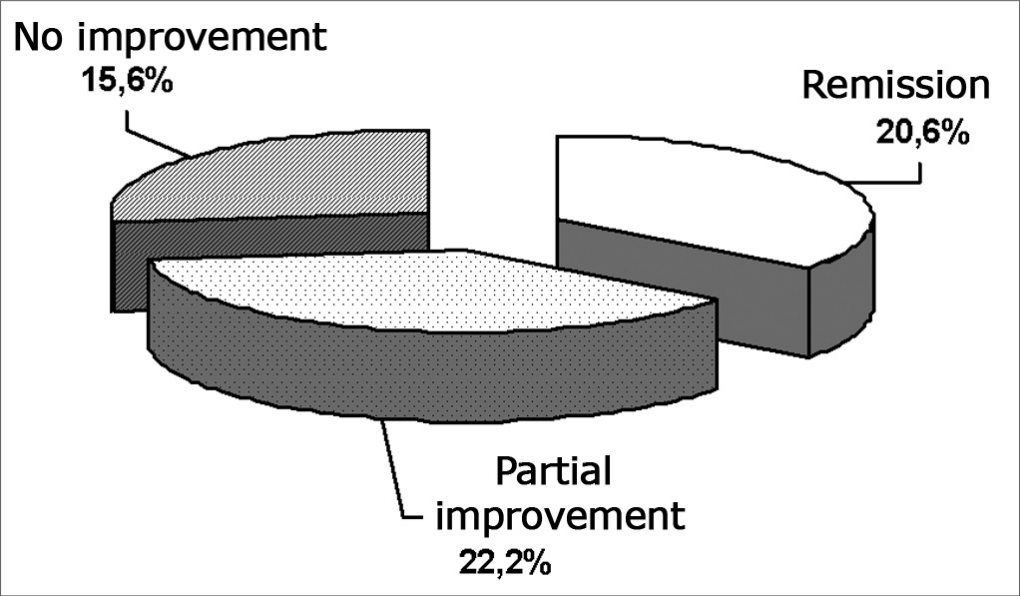
Distribution of the total sample in function of the VR program results.

Between 2002 and 2004 we treated 243 patients with VR. Of these, 68 (28%) abandoned the treatment, 33 (13.6%) had their treatment suspended for varied reasons, and 142 (58.4%) concluded their therapy protocols. If we consider that of the 142 who concluded VR, 50 (35.2%) had remission of all symptoms, 54 (38%) had only partial improvement and 38 (26.8%) belonged to the no improvement group. Graph 1 shows the distribution of the total number of patients who started VR and their results.

The 142 patients had their charts carefully reviewed. We then selected 59 (41.5%) patients who had confirmed etiology according to the predicted diagnostic and including criteria. Therefore, in our final sample we had 13 patients who did not benefit from VR (no improvement), 24 with partial improvement and 22 with symptoms remission. Observed mean age was 54.5 years with a standard deviation (SD) of 15.8.

When we analyzed the 59 patients included, we noticed that the most frequent etiologies were: neck segment involvement, trauma (iatrogenic or accidental) and metabolism disorders, which together responded for 76.3% of the cases. We also observed central injuries in 13.6% of the cases, MSD in 5%, OI in 3.4% and AID in 1.7%. Table 1 shows the final etiological diagnoses and its percentages.

**Table 1:** Diziness etiology diagnosis in relation to the VR results in the many groups.

		Groups		
Etiology	No improvement	Partial improvement	Remission	Total
Central	3	3	2	8 (13.5%)
Trauma	3	6	4	13 (22.1%)
Metabolic	2	6	12	20 (33.9%)
MSD	2	1	0	3 (5.1%)
Cervical	2	7	3	12 (20.3%)
OI	0	1	1	2 (3.4%)
AID	1	0	0	1 (1.7%)
Total	13 (22.1%)	24 (40.7%)	22 (37.3%)	59 (100%)

MSD: mood and stress disorder; OI: orthostatic intolerance; AID: autoimmune disorder.

In the group that had remission, the four cases of trauma (100%) happened after otologic surgery (mastoidectomy, stapedotomy, vestibular schwannoma exeresis), in the partial improvement two cases are post-operative (50%) and in the no improvement group, one patient (30%) came from surgery.

**Graph 2:**
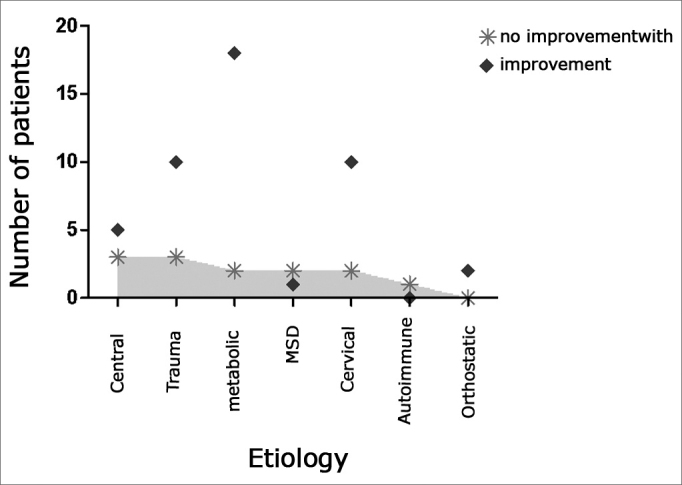
Most prevalent etiologies among treatment groups.

In order to better appreciate VR results, Graph 2 shows the scattering of values obtained from the different etiologies. Results were broken down in no improvement or improvement (corresponding to the total number of patients who had remission or partial improvement).

Observing the scatter graph (Graph 2), we see that in the groups encompassing MSD and AID the number of dissatisfied responses supersedes the number of cases who had improvement from VR treatment.

Central etiology did not show improvement in 37.5% of the cases treated, while all the patients together did not respond to treatment in 22.1% of the time. It was not possible to establish a statistical association because of the few cases we had.

Three diagnostics were present in a sufficient number for individual assessment: metabolic, neck and trauma. The respective answers to VR treatment were compared individually in relation to the response from the whole group. The group classified as metabolic presented a significantly better response (p=0.029) when compared to the other patients, while diagnostics of neck (p=0.375) and trauma (p=0.851) did not show variation in relation to the other groups in the sample.

## DISCUSSION

With the progress of knowledge regarding the mechanisms involved in VR, it started to be used in cases that were previously contra-indicated, such as in MD8. In this case, its final goal is not vestibular adaptation, but rather patient orientation, explaining the symptoms and teaching them how to use strategies to help face the crisis, thus reducing the stress related to the disease. Thus, VR can be seen as a multimodal therapy, which will be planned according to the etiology of diziness and the individual needs of each patient.

In the case of vestibular diseases of metabolic origin, we should use the same principles used for MD, because often times the substrate is hydrops. VR will be very efficient as long as the disorder that caused the vestibular dysfunction is treated, being it glucose-related, thyroidal or lipids^9^. We know the influence of comorbidities in the final response to VR treatment^13^. When not properly solved, systemic processes end up negatively influencing vestibular adaptation, causing a partial response or even no response at all. In our series, we observed that the metabolic etiology was prevalent among the groups that showed better response to VR, and this happens because the patients were rehabilitated together with the correction of systemic disorders.

In this study, we considered the cervical syndrome as a functional process of propioceptive origin, that may or may not be associated with structural lesions^12^. Morning feeling of unbalance is the major complaint. The local treatment for the cervical region is a fundamental factor in the recovery of these patients, which can have symptoms melioration with proper physical therapy. Although we have not observed statistical difference in evolution in relation to the other groups, the percentage of patients who presented partial improvement was greater than the individuals who had symptoms remission. This observation suggests that VR as a single therapy is not sufficient to resolve the propioceptive cases entirely. We have observed good results in neck problems, because we associated VR with physical therapy14. In these cases, physical therapy is fundamental in approaching the physiological process, being efficient in solving pain-related symptoms.

Of special interest is the result attained in the group that suffered injuries, in that group we gathered the cases of otologic surgery, both in the inner and middle ear, followed by vertigo. We can see that from the entire group with remission, 50% that had partial improvement and 30% that had no improvement had been submitted to ear surgeries. These cases portray one of the most satisfactory results of VR, that the earlier it is started, the more benefit it will provide in terms of therapeutic results^15^.

In a previous publication we observed that the patient with central nervous system involvement is the one who presented the worst response to treatment, representing approximately 50% of the cases1. According to this survey, the syndromes of central etiology represented the number one cause of limited response to VR. In accordance to other authors, our experience shows that cerebellar lesions, although they respond to VR, are the ones bearing the worst prognosis among central lesions^11^. Here there is a basic caveat: VR is eventually the only treatment with some beneficial effect to these patients, often times bedridden. Although we do not expect a resolution of the body balance problems, VR improves balance problems and is highly beneficial for these individuals.

When we look at the scatter graph (Graph 2), we find a larger number of individuals who do not respond well to VR when compared to those that do respond in the group of patients with mood and anxiety disorders. It is an interesting aspect, because it indicates that VR does not work as a placebo therapy in solving the symptoms of these patients. The symptoms of a panic spell are very similar to those of a labyrinth origin and their association has been well established in the literature16. These crises are usually related to some specific situation, but they can occur alone. Thus, often times diagnosis is only made by the strategic use of VR, which here can work also as a diagnostic tool. Cases of anxiety, panic and depression do not improve with VR and must be treated with specific medication or psychotherapy.

As far as IAD is concerned, we must stress that they usually evolve in spells and follow the same guidelines of metabolic disorders. VR can be used as support and guidance, and it should not be expected that its deployment alone will improve the patient's symptoms. By observing the scatter graph, we see the only patient in the sample who did not benefit from the treatment.

As far as OI is concerned, diziness is characteristically associated with decubitus. Like OI, cardiogenic, neurologic and reflex-mediated origins are associated with diziness that happens when the patient stands up and must be ruled out17. OI may be associated with the excessive use of anti-hypertensive medication and is relatively common in the elderly. In both cases there is no indication for VR, but rather of titrating the drug dosage or start physical exercises that strengthen lower limbs' muscles.

## CONCLUSION

We reiterate the value of VR in the treatment of vestibular disorders. As an ancillary or single treatment, its prognosis depends on the etiology of diziness.

## References

[1] Bittar RS, Pedalini ME, Lorenzi MC, Formigoni LG (2002). Treating vertigo with vestibular rehabilitation: results in 155 patients.. Rev Laryngol Otol Rhinol (Bord).

[2] Honrubia V, Bell TS, Harris MR Baloh RW, Fisher LN (1996). Quantitative evaluation of dizziness characteristics and impact on quality of life.. Am J Otol.

[3] Ganança FF, Castro ASO, Branco FC, Natour J (2004). Interferência da tontura na qualidade de vida de pacientes com síndrome vestibular periférica.. Rev Bras Otorrinolaringol.

[4] Nishino LK, Gananca CF, Manso A, Campos CA, Korn GP (2005). Personalized vestibular rehabilitation: medical chart survey with patients seen at the ambulatory of otoneurology of I.S.C.M.S.P.. Rev Bras Otorrinolaringol (Engl Ed).

[5] Kenyon RV, Leigh J, Keshner EA (2004). Considerations for the future development of virtual technology as a rehabilitation tool.. J Neuroengineering Rehabil.

[6] Sparto PJ, Whitney SL, Hodges LF, Furman JM, Redfern MS (2004). Simulator sickness when performing gaze shifts within a wide field of view optic flow environment: preliminary evidence for using virtual reality in vestibular rehabilitation.. J Neuroengineering Rehabil.

[7] Medeiros IR, Bittar RS, Pedalini ME, Lorenzi MC, Formigoni LG, Bento RF (2005). Vestibular rehabilitation therapy in children.. Otol Neurotol.

[8] Dowdal-Osborn M (2002). Early vestibular rehabilitation in patients with Menieres disease.. Otolaryngol Clin North Am.

[9] Bittar RSM, Bottino MA, Zerati FE, Moraes CLO, Cunha AU, Bento RF (2003). Prevalency of metabolic disorders in dizzy patients.. Rev Bras Otorrinolaringol (Engl Ed).

[10] Pedalini MEB, Bittar RSM (1999). Reabilitação vestibular: uma proposta de trabalho.. Pró-fono.

[11] Brown KE, Whitney SL, Marchetti GF, Wrisley DM, Furman JM (2006). Physical therapy for central vestibular dysfunction.. Arch Phys Med Rehabil.

[12] Brandt T, Bronstein AM (2001). Cervical vertigo.. J Neurol Neurosurg Psychiatry.

[13] Cohen HS, Kimball KT, Stewart MG (2004). Bening paroxysmal positional vertigo and comorbid conditions.. ORL J Otorhinolaryngol Relat Spec.

[15] Herdman SJ, Clendaniel RA, Mattox DE, Holliday MJ, Niparko JK (1995). Vestibular adaptation exercises and recovery: acute stage after acoustic neuroma resection.. Otolaryngol Head Neck Surg.

